# Sinonasal Malignancies and Charged Particle Radiation Treatment: A Systematic Literature Review

**DOI:** 10.1155/2012/325891

**Published:** 2012-05-27

**Authors:** Marco Cianchetti, Maurizio Amichetti

**Affiliations:** Agenzia Provinciale per la Protonterapia Trento (ATrep), Via Fratelli Perini, 181, 38122 Trento, Italy

## Abstract

*Background*. Paranasal and nasal cavity malignancies are rare tumors that frequently present at advanced stages. Tumor extension and anatomic complexity pose a challenge for their treatment. Due to their peculiar physical and biological properties particle radiation therapy, i.e. protons and ions can have a role in their management. We performed a systematic literature review to gather clinical evidence about their use to treat sinonasal malignancies. *Materials and Methods*. We searched the browsers PubMed and Medline as well as specific journals and conference proceedings. Inclusion criteria were: at least 10 patients, English language, reporting outcome and/or toxicity data. *Results*. We found six studies with data on clinical outcome. Carbon and helium ions were each used in one study, protons in four. Toxicity was specifically described in five studies. One reported acute toxicity of carbon ions, one dealt with brain toxicity from both carbon ions and protons. Three papers reported on visual toxicity: one from carbon ions, one from protons and one from both. Specific data were extracted and compared with the most pertinent literature. *Conclusion*. Particle radiation therapy is in its early phase of development. Promising results achieved so far must be confirmed in further studies.

## 1. Introduction

Paranasal sinus and nasal cavity malignancies are rare with an incidence rate estimated to range from 0.3 to 3.5 per 100.000 per annum [1, 2]. They account for 3% of head and neck carcinomas and about 0.5% of all malignant diseases [3]. Squamous cell carcinoma is the most common histology; however, adenocarcinoma, adenoid cystic carcinoma, and undifferentiated carcinoma are relatively common too [4]. Several other histotypes like malignant melanoma [5], sarcoma [6], neuroendocrine tumors, namely, esthesioneuroblastoma, sinonasal undifferentiated carcinoma, neuroendocrine carcinoma, and small cell undifferentiated carcinoma [7, 8] can occur but are uncommon. Different incidence rates and pathology distribution are observed in different geographical areas and they are thought to relate mainly to professional exposure [1, 2] such as among wood and furniture [1, 2, 9] workers. Males are usually more frequently affected than females [1].

Sinonasal malignancies present frequently at advanced stages due to late symptom onset. This, combined with the complex regional anatomy and the presence of several organs at risk (OARs) such as brain and optic structures, poses a challenge for their best management. The mainstay of treatment is surgery [10]: traditionally a craniofacial approach [11–13] has been employed. In recent years, in order to achieve wider resection margins and or to spare significant side effects, craniotomy and endoscopic approaches or their combination have been tested with encouraging results [14–16].

 Radiation therapy has been employed either as adjuvant treatment for high-risk cases or as definitive therapy for unresectable disease. No clear evidence exists for a routine use of chemotherapy, which is administered generally in a case-by-case scenario [17, 18]. Despite the therapeutic efforts, results are not completely satisfactory, with overall survival rates at 5 years ranging from 50% to 67% [19, 20] for combined treatment and from 15% to 38% when radiation therapy is given as the sole treatment [10, 21]. Furthermore, side effects associated with the treatment can be significant [22, 23]. The use of highly conformal techniques has demonstrated its feasibility even in advanced stages and has shown a significant reduction of side effects [24].

**Figure 1:**
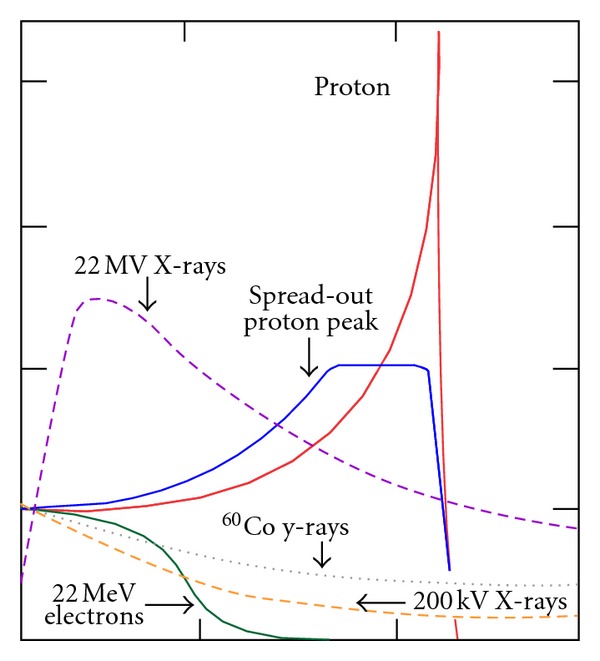
Bragg peak and Spread-Out Bragg Peak (SOBP) for a proton beam in comparison with photon and electron dose distributions.

Particle radiation therapy, that is, protons and heavy ions, is a relatively new type of radiation therapy that could enhance the therapeutic ratio for sinonasal malignancies. Protons and heavy ions share the same characteristic dose distribution, the so-called Bragg Peak, that is the release of almost all their energy in a few millimeters at the end of their path (see Figure 1). This peak is narrow and not suitable to treat target volumes so it has to be opportunely spread out, the so-called SOBP-Spread Out-Bragg Peak, allowing thus the delivery of high doses sparing at the same time OARs. Comparative treatment planning studies have demonstrated the robustness of heavy particle generated plans and showed their advantages over photon plans [25]. Protons have a relative biologic effectiveness (RBE) that is slightly higher than photons according to some authors [26], its value being around 1.1, or exactly the same as photons according to others [27]. Anyhow, the International Commission on Radiation Units and Measurements (ICRU) in its 78 report issued in 2007 recommends using a value of 1.1 [28]. More complex is the situation for heavy charged particles since the RBE is not constant along their path, but increases with increasing depth, reaching the maximum at the peak region [29]. RBE is dependent indeed by the microdosimetric pattern of energy deposition that is, the Linear Energy Transfer (LET) that increases as they slow down [30]. The advantage of high LET radiations is that there is less variation in radiosensitivity during the cell cycle, less reduction of repair of radiation injury, and lower oxygen enhancement ratio [31]. From preparatory studies, an RBE value of 3 for carbon ion was found at the distal part of the SOPB [32]. In order of taking into account the different RBE of particle radiation therapy, their dose is expressed in Gy (RBE) that is the product of the physical dose in Gy multiplied for the specific RBE [30].

An altered fractionation regimen is any radiotherapy schedule that differs from the standard delivery of 1.8–2.0 Gy, 5 days a week for an overall treatment time of about 6-7 weeks. They can be classified as hyperfractionated, accelerated, or hypofractionated with possible combinations as well. They all try to increase the therapeutic index that is, the ratio of the probability of tumor control to the probability of normal tissue toxicity. Published papers in the head and neck field have proved their effectiveness in comparison to standard fractionation [33, 34]. Particle radiation therapy has been delivered as well with altered fractionation schemes taking advantage of its particular physical and biological properties [35, 36]. When comparing studies that employed different fractionation regimes, it is useful to refer to the Biologically Effective Dose (BED). This mathematical formula, BED = *nd*(1 + *d*/*α*/*β*), [37], allows comparison of the toxicity or efficacy of different fractionation regimes knowing the number of fractions (*n*), the single dose (*d*), and the **α*/*β** ratio that is a radiobiological parameter that characterizes the response of every tissue to radiations.

Since particle radiation therapy is not very widespread and basically still under development, data of its use to treat sinonasal malignancies are scant.

In this paper we performed a systematic review of the literature to gather all the clinical experience so far accumulated on this issue focusing on outcome and side effects.

## 2. Materials and Methods

The Population-Interventions-Comparators-Outcomes (PICO) [38] framework was used to find appropriate keywords used to search the databases. Free text words and Mesh terms for paranasal sinus malignancies or particle radiation therapy or outcome or toxicity were generated and then combined according to Boolean operation “AND” or “OR.” A research of the literature was carried out in PUBMED and MEDLINE first in August 2011 and then updated in October 2011. Manual search of the bibliography of pertinent papers was performed too. Any study known by any author was also considered for inclusion.

Eligibility criteria were as follows: studies published in the English language, reporting outcome and/or toxicity data on definitive treatment with particle radiation treatment of nasal and paranasal sinus malignancies. Studies were accepted if photon radiation therapy was combined with particle radiation and if radiation therapy was used either in definitive, adjuvant, or neoadjuvant setting. Any use of chemotherapy was allowed. Studies had to include at least 10 patients and to report data on tumor control and/or on toxicity. Studies including multiple head and neck sites were considered and included if it was possible to extract specific information on sinonasal malignancies. In case of publications with overlapping data, the study with the largest number and wider data was chosen. Review articles, case reports, and planning studies were not considered.

Data extracted from publications meeting the eligibility criteria were first author, year of publication, institution, number of patients, gender, median age, pathology, stage, type of surgery received if any, type of chemotherapy received if any, and followup. Regarding radiation treatment, the following data were extracted: possible combination with photon, type of particle used, RBE employed, total dose, dose per fraction, number of fractions and number of fractions a week of the particle used, total dose, dose per fraction, number of fraction and number of fractions a week of photons; combined total dose was reported as well. For studies reporting results on tumor control the following data were recorded: local, regional and distant control, overall survival, type, scale and grade of toxicity, time to toxicity, and risk factors individuated. For studies dealing particularly with toxicity apart from the available data as above, the diagnostic criteria used were reported as well as the maximum dose to affected organs and side effect risk factors. Any toxicity scale used was also reported.

## 3. Results

**Figure 2:**
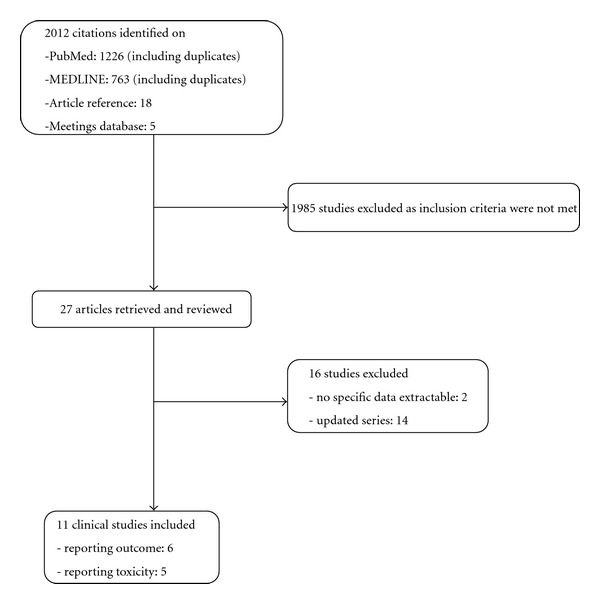
Flowchart of the searching process.

The initial literature research identified 2012 studies including duplicates (see Figure 2). 1985 were excluded after reading the title or the abstract, as they did not fulfill the inclusion criteria. Twenty-seven studies remained and were considered for inclusion. After thorough reading of the full text, two studies were excluded as specific data of sinonasal malignancies were not available and fourteen were also excluded as they have subsequently been updated or because their data had already been included in other papers with larger numbers. At the end of the searching process, we identified six studies reporting outcome data (see Tables 1, 2, 3) of which two dealt with ions and four with protons. Five studies reporting exclusively toxicity were also included, two on ions, one on protons, and two on both (see Tables 4, 5).

### 3.1. Studies Reporting Outcome Data

All the studies reporting data on outcome have been published in peer-reviewed journals apart from the one from Malayapa [39] that has been presented at the 52nd annual meeting of the American Society for Radiation Oncology and published in the relative proceedings.

The first publication included in the paper, by Castro et al. [40], dates back to 1994 and reports results from “The University of California—Lawrence Berkeley Laboratory.” Even though the paper deals with skull base malignancies, specific data on 22 paranasal and nasal cavity malignancies were available. All the patients received combined photon and helium ions radiotherapy for their tumor extending to the cranial base. Energies used in the synchrocyclotron were 215/232 MeV/u; RBE was 1.3 except for central nervous system where it was considered to be 1.6. Total dose ranged from 60 to 80 Gy (RBE), median 65 Gy (RBE). Further details on general treatment were not reported. Five-year actuarial local control and overall survival were 60% and 38%, respectively. Data on toxicity are difficult to extract since they refer to a wider group of patients with other malignancies and treated also with neon ions. It would seem anyhow that the largest amount of toxicity is related to the use of neon ions.

Mizoe et al. [31] published the results from the NIRS (National Institute of Radiological Sciences), Heavy Ion Medical Accelerator in Chiba, Japan. Since 1994, they have been using carbon ions because of their biologic and physical characteristics. Carbon ions' LET and RBE increase with depth reaching the maximum at the Bragg Peak: here they show an RBE of 3.0 if compared to photons [41]. Furthermore, experiments have demonstrated that increasing the carbon ion dose fraction tends to lower the RBE both for tumor and normal tissues, but more pronouncedly for the latter [35]. Hypofractionation schemes can be, therefore, used in carbon ion radiotherapy to increase the therapeutic ratio. Mizoe et al. [31] report the results of two dose escalation clinical studies for patients with multiple head and neck sites. Patients were treated, respectively, according to a schedule based on 18 fractions in three fractions a week for six weeks (Group A) or to another based on 16 fractions for four weeks in four fractions a week (Group B). Doses for each group were escalated every three to five patients, in 10% increments starting from 48.6 Gy (RBE) for Group A and from 52.8 Gy (RBE) for Group B. Median total dose for Group A was 59.4 Gy (RBE) (range: 48.6–70.2 Gy (RBE)) and 57.6 Gy (RBE) (range: 52.8–64.0 Gy (RBE)) for group B. No photons were employed. Acute reactions were scored according to the RTOG toxicity criteria system, late to RTOG/EORTC. The Kaplan-Meier local control for both groups was 49% at five years. Toxicity was reported for the entire cohort and no specific data on sinonasal tumors were retrievable. Acute Grade (G) 3 skin toxicity was seen in one Group A patient and in six Group B patients; G3 mucous membrane toxicity just in one Group B patient. No greater than G3 toxicity was observed.

Tokuuye et al. [42] from the University of Tsukuba, Proton Medical Research Center (PMRC), Japan, reported data of eleven patients with sinonasal malignancies, treated with protons (four) or with a combination of photons and protons (seven). They all had inoperable disease or refused surgery. Median total dose delivered was 72 Gy (RBE) (range: 42–98 Gy (RBE)). RBE value was 1.1. Local control was achieved in nine out of eleven patients and at the moment of the analysis five out of eleven patients were alive. One case of osteoradionecrosis was observed. Again, no specific sinonasal toxicity data were distinguishable from that of other head and neck sites. In the toxicity analysis a trend for BED_(*α*/*β*=3)_ > 130 Gy (RBE) was associated with late toxicity as well as with high fraction size.

Data from Massachusetts General Hospital, Harvard Cyclotron Laboratory, Francis Burr Proton Center, USA, are available in the paper of Resto et al. [43] From 1991 to 2002, 102 patients with locally advanced sinonasal malignancies of various histology received a mixed photon/proton treatment. Radiotherapy was given as definitive treatment in 32 cases, as adjuvant after partial resection in 50, and as adjuvant after complete resection in 20. Median total dose was 71.6 Gy (RBE); median percentage of protons was 57.1%. Twice-a-day fractionation schemes were employed in 80% of the cases. Local control was 95%, 82%, and 87%, respectively, for patients with complete resection, partial resection, and biopsy only. Analogously overall survival was 90%, 53%, and 49%, respectively. Regional control and distant metastasis-free survival were, respectively, 88% and 69%.

Zenda et al. [44] from the National Cancer Center Hospital East, Chiba Japan, reviewed their results for 39 patients treated with proton radiation therapy. They all had T4N0M0 unresectable disease of various pathologic types. A full course of proton therapy, RBE 1.1, was delivered to a median total dose of 65 Gy (RBE) with a median dose per fraction of  2.5 Gy (RBE). Actuarial one-year local control and five-year overall survival were 77.0% and 55%, respectively. Severe acute toxicity was not observed. Late toxicity G3–5 was reported in 5 patients (12.8%), G5 in 1 patient (2.6%) who suffered a CSF leakage.

The University Of Florida Proton Therapy (UFPT) Institute's experience is reported in the paper of Malyapa et al. [39] since January 2007, they have treated 38 patients with various histotypes of sinonasal malignancies of which three were in the definitive setting and 35 in the adjuvant setting. Positive margins were present in 17 cases of the latter. Maximum total doses for patients with negative margins ranged between 68.8 and 69.9 Gy (RBE), maximum dose for patients with positive margins or unresectable disease was 74.4 Gy (RBE). In every case, a twice-a-day fractionation regime was employed: single dose was 1.2 Gy (RBE) with fractions separated each other at least by six hours. At the moment of the analysis, two in-field progressions, one meningeal seeding, and three distant metastases were observed. One case of retinopathy within the treated volume occurred without significantly impairing acuity vision.

### 3.2. Studies Reporting Toxicity Data

A total of five studies dealing specifically with toxicity after particle radiation therapy were found. Jensen et al. [45] from the University of Heidelberg, documented exclusively acute toxicity for 29 patients with T4 sinonasal malignancy treated with carbon ion plus or minus photon radiotherapy to a median dose of 73 Gy (RBE). Accrual period was from November 2009 to August 2010 during which 17 patients received definitive treatment, two reirradiations, and nine radiotherapy after local relapse. Carbon ion total dose was 24 Gy (RBE) delivered in fractions of 3 Gy (RBE); IMRT total dose was 50 Gy in 25 fractions. Acute toxicity was scored according to CTCAE versus 4.0. There were no G4 or G5 toxicities and toxicity related interruption. Mucositis and dysphagia G3 occurred five and two times, respectively. 

Demizu et al. [46], from the Hyogo Ion Beam Medical Center, Japan, described side effects for 104 patients treated with carbon ions or protons for their tumor adjacent to optic nerves. Nasal cavity or paranasal sinuses were involved in 52 cases of which 10 (19.2%) received carbon ions and 42 (80.8%) proton therapy. Carbon ion total dose was 57.6 Gy (RBE) in 16 fractions; proton total dose was 65 Gy (RBE) in 18 fractions. Both treatments were administrated five days a week. Visual loss resulting from optic neuropathy was seen at 52 months in one (10%) patient who had received carbon ions and at a median of 34.5 months in four patients (9.5%) that had received protons. At univariate analysis age >60 years, diabetes mellitus and BED_max⁡_ > 110 Gy (RBE) (*α*/*β* = 3) were observed as possible risk factors. Diabetes was statistically significant at multivariate analysis too. 

In the paper of Hasegawa et al. [47], from the National Institute of Radiological Sciences (NIRS) in Chiba, Japan, 14 patients with paranasal or nasal malignancies that received proton radiation treatment are included. It was not possible to retrieve details on other treatments received since they are described with those of patients with other head and neck malignancies. Significant visual loss was reported in seven patients (50%), for whom optic nerve sparing had been difficult due to tumor involvement. Risk factors for the entire group at univariate analysis were male gender, chemotherapy, anemia, diabetes mellitus, prescribed tumor dose, D_max_ of the optic nerve >57 Gy (RBE), and D_10-50_. At multivariate analysis, D_20 _> 60 Gy (RBE) was still significant. 

Weber et al. [48] described the visual outcome for patients treated with accelerated combined proton-photon radiotherapy for advanced sinonasal malignancies at Massachusetts General Hospital, Harvard Cyclotron Laboratory, Francis Burr Proton Center from 1991 to 2001. Given the demonstrated higher efficacy of altered fractionation regimens towards head and neck malignancies [33], a median dose of 69.6 Gy (RBE) was given to 36 patients twice a day, with a median fraction size of 1.8 Gy (RBE) for protons and 1.6 Gy for photons. Percentage of cases treated with protons ranged between 20% and 84.4%. Complications of the optic nerves, retina, and lenses were scored according to CTCAE versus 2.0 scale, of all the others visual organs to LENT. Grade 3 late toxicity occurred in 2 patients (8.3%), G2 in 6 (16.7%) and G1 in 5 (13.9%). No retinopathy or optic nerve damage higher than G1 was observed. The reported 5-year probability of higher than G2 toxicity was 20.7% ± 7.8%. Risk factors were GTV dose, age, D50 and D90 to the optic chiasm, and D10 and D50 to the optic nerve. No association was found between tumor localization in respect to the optical apparatus and toxicity. 

The experience of The Hyogo Ion Beam Medical Center about radiation-induced brain injury after proton or carbon ions is reported in the paper of Miyawaki et al. [49]. Twenty-eight patients with sinonasal malignancies, out of fifty-nine of the whole group with head and neck cancers, received particle radiation therapy either as carbon ions, five, or protons, twenty-three. The total dose for the former group was 57.6 Gy (RBE) in 16 fractions of 3.6 Gy (RBE), for the latter was 65 Gy (RBE) in 26 fractions of 2.5 Gy (RBE). To compare different fractionation schedules they used a biologically effective dose with *α*/*β* value of 3.6. Radiation-induced brain changes (RIBCs) were evaluated by MRI findings on T2-weighted or postcontrast images and graded according to the LENT-SOMA scale. Related symptoms were scored according to CTCAE versus 3.0 scale. After a median interval of 31 (range: 6–49) and 27.5 (range: 19–36) months, respectively, three patients, treated by protons, and two by carbon ions developed radiation-induced brain changes. Radiologic toxicity grades for the three protons-treated patients were G1, G2, and G3 and for the two carbon ions-treated patients were G3 and G2. For protons-treated patients, clinical grading was G1 for one and G2 for two, meanwhile it was G3 and G1 for the two carbon ions-treated patients. G3 clinical toxicity consisted of epilepsy requiring steroids and anticonvulsants. From the analysis of the whole group, carbon ions were statistically more frequently associated with RIBCs than protons (*P* = 0.02). Minimal median dose to RIBCs sites for the three proton patients was 117.1 Gy (RBE)_3_ (range: 59.4–117.1 Gy (RBE)) and 27.5 Gy (RBE)_3_ (range: 102.4–110.6 Gy (RBE)_3_) for the two receiving carbon ions. Most of the RIBCs were induced by doses ≥80 Gy (RBE)_3_, occurring within two years from radiation in comparison to those induced by lower doses that developed after two years. Lobe volumes receiving more than 83, 90, and 100 Gy (RBE)_3_ were significantly associated with RIBCs. 

## 4. Discussion

We were able to find 11 studies reporting outcome and/or toxicity results relative to the use of particle radiation therapy for the treatment of paranasal sinus and nasal cavity malignancies. There are some common limitations for these studies that must be emphasized. All but one study (Mizoe et al. [31]) were retrospective. Six of them reported data on multiple head and neck sites, and it was necessary to extract specific numbers for sinonasal malignancies. Moreover, they included few patients accrued over a long period of time. Some studies have a short follow-up period. Not all the treatments were delivered in dedicated facilities so that logistic hurdles had to be overcome in this case [40, 42]. Frequently, various pathologic types are grouped together even if it is well known that response to treatment varies among them [4, 20]. Lastly, it was not always possible to retrieve all the details regarding patients' general management. Nonetheless, some conclusions from the available data can be made. 

Heavy particle radiotherapy is in its early phase of development, and it is mainly employed for unresectable or high-risk cases. This type of radiation therapy combined with photons or not is feasible and well tolerated by patients either as definitive or as adjuvant treatment. The delivery of the prescribed dose was possible in all studies, and all patients could complete their treatment. Altered fractionation schemes were safely employed to increase the therapeutic ratio [31, 39, 44, 48], but other studies [42, 49] found that late radiation toxicity can be related to the high single fraction dose. 

Local control rates compare positively with those from Institutions that used photon radiation therapy (Table 6). Local control rates at 5 years achieved with conformal photon radiotherapy are around 62%–84% [20, 52] for the postoperative setting and around 21%–43% [10, 21] for definitive treatment. Resto et al. [43] showed local control rates at 5 years according to extent of resection that were 95%, 82%, and 87% for complete resection, partial resection, and biopsy only, respectively. At univariate analysis, these figures did not differ significantly (*P* = 0.32). Castro et al. [40] reported a 5 year local control rate of 60%; Zenda et al. [44] had one-year local control of 77%, for 39 patients with locally advanced disease. Malyapa et al. [39] at the moment of the analysis reported that 36 out of 38 patients were free of local disease; for Tokuuye et al. [42], 9 out of 11 patients were free of local recurrence. The apparently lower local control rate from Mizoe et al. [31] must be taken into account cautiously considering that it was a Phase I/II study whose main purpose was to determine the normal tissue tolerance dose. Indeed some of the patients were given a dose that was thought to be 80% of the radical dose needed for advanced head and neck cancers. Interestingly, in the study of Resto et al. [43] local control was found to be independent from the extent of resection. Rates of distant metastasis free-survival and overall survival were significantly different in case of complete resection, 95% and 90%, respectively, versus partial, 69% and 53%, respectively, or biopsy only, 52% and 49%, respectively (*P* = 0.03, *P* = 0.02). 

In general, for particle radiation therapy studies, rates of distant metastasis free-survival and overall survival are not substantially different from other studies. Obviously, this means that an efficient systemic therapy capable of dealing with metastasis is needed for particle therapy too. 

Regional control is reported in the papers of Resto et al. [43] and Zenda et al. [44] only. Details of neck treatment are not reported extensively; regional control rates are similar being 88% and 87.2%, respectively, confirming that regional failures are quite infrequent in this disease. 

Acute toxicity seems to be mild, well tolerated, and does not interfere with treatment delivery as pointed out in the specific paper by Jensen et al. [45]. Late toxicity was not reported extensively by all studies of the group reporting data on outcome. Mizoe et al. [31] reported no late toxicity; Tokuuye et al. [42] reported just one case of osteoradionecrosis. The only G5 toxicity was described by Zenda et al. [44], which was due to CSF leakage. Late toxicity was specifically reviewed in four papers, three of them dealing with visual side effects and one with CNS side effects. Median time to the onset of visual side effects ranged between 24 and 52 months. In the study of Weber et al. [48], higher doses were associated with faster development of side effects, analogously to what already described by other authors [53]. Radiation-induced optic neuropathy leading to severe visual loss occurred in five out of fifty-two (9.6%) and in seven out of fourteen (50%) patients for the studies of Demizu et al. [46] and Hasegawa et al. [47], respectively. The latter figure may seem high, but it is to note that all the optic nerves showing high-grade toxicity had been invaded by the tumor making it almost impossible to spare them. The negative effect of the high RBE of carbon ions cannot be completely ruled out though. The result by Demizu et al. [46] seems acceptable considering the particular location of disease and when compared to other data. Jiang et al. [54] reported blindness due to optic neuropathy in 8 out of 98 (8.1%) evaluable patients who had received radiation therapy for sinonasal malignancies. In a previous publication from Massachusetts General Hospital, Habrand et al. [55] reported data on neurovisual outcome after proton radiotherapy for upper clivus malignancies; two patients out of fifteen (13.3%) developed visual deterioration at 10 and 36 months, respectively. This was due to optic nerve and optic chiasm damage in one and to bilateral optic nerve damage in the other. Results from the study of Weber et al. [48] are encouraging, being that any type of visual toxicity ≥G2 is around 20%. In fact, they only had two G3 complications out of thirty-six patients (8.3%), namely, a cataract and a nasolacrimal duct blockage. Jiang et al. [54] described a total of 60 ipsilateral visual events in 178 patients treated (33.8%); seven of them experienced also contralateral visual impairment. 

Among the possible risk factors, all the studies of particle radiation therapy found important the maximum dose delivered. This confirms both the concept that many optic structures are serial organs and the results from previous studies. Parsons et al. [22] observed indeed no injuries in 131 patients with head and neck malignancies if their optic nerves were irradiated to less than 59 Gy. Jiang et al. [54] in their study about 219 individuals with cancers of the nasal cavity and paranasal sinuses found that the radiation dose was the predominant determinant for optic nerves and optic chiasm injury with no patient receiving less than 50 Gy showing any impairment. Analogously, Takeda et al. [56] did not observe any retinal complications for patients receiving less than 50 Gy. Habrand et al. [55] suggested a threshold value of 55 Gy (RBE) associated with a 10% major complication rate. Urie et al. [57] found as well that the dose is a significant predictor of cranial nerve injury but with a value of 70 Gy (RBE) that is higher than what is commonly reported. 

A volume factor for the irradiated OAR has been individuated as risk factor in the study of Hasegawa et al. [47]. The dose to the 20% of the optic nerve higher than 60 Gy (RBE) was significantly associated with visual loss at multivariate analysis. This is in accordance with the findings of Weber et al. [48] that reported partial OAR volume irradiation as a risk factor even if they pointed out that setup uncertainties and contouring variability may confound data. Takeda et al. [56] found as well that irradiated retina area correlates with the incidence of sever late complication. 

Dry-eye syndrome is a serious side effect associated with head and neck radiotherapy [53]. In this regard, the study of Weber et al. [48] showed a low incidence rate of serious toxicity since they were able to deliver to the lachrymal apparatus a dose below the generally accepted threshold value of 30 Gy [53]. Furthermore, their good results may be related to the use of a twice-a-day fractionation schedule. Monroe et al. [58] studying 186 patients with head and neck malignancies found that a twice-a-day hyperfractionation schedule with single doses between 1.1 and 1.2 Gy, separated at least by 4 h–6 h, was significantly associated with lower incidence of retinopathy. The same group (Bhandare et al. [59]) observed that also optic neuropathy may be reduced with the same schema. This is in accordance also with the report of Malyapa et al. [39] that described just one case of mild retinopathy for a group of patients treated with hyperfractionated proton therapy. As regards to hypofractionation, Parsons et al. [60] showed that the use of larger fraction size is associated with retinal toxicity. In the study of Demizu et al. [46], single fraction was 3.6 Gy (RBE) for carbon ions and 2.5 Gy (RBE) for protons and they did not raise particular concern about the utilization of high-dose per fraction. 

Other risk factors found by the specific studies were diabetes mellitus that has already been associated with toxicity by other authors [59, 61] and age where data from published experience are not definitive [22, 58, 59]. The study of Weber et al. [48] has the apparently paradox finding that younger age is linked with higher toxicity. This may be explained with the longer follow-up period elapsed for this group of patients. Data on the use of chemotherapy are not definitive: it is positively related to toxicity at univariate analysis in the study of Hasegawa et al. [47] but not for Weber et al. [48], Bhandare et al. [59], and Takeda et al. [56]. 

Brain late side effects have always been among the most dreaded consequences of radiation therapy. Significant neurologic alterations have been described in association with photon and particle radiotherapy for paranasal and nasal cavity malignancies [23, 62]. The study of Miyawaki et al. [49] has a good toxicity profile with just one patient out of 28 experiencing G3 brain toxicity, that is, epilepsy requiring medical treatment. Factors related to toxicity were dose ≥80 Gy (RBE)_3_ and volumes of cerebral lobe receiving more than 83, 90, and 100 Gy (RBE)_3_. Maximum dose and volume receiving a given dose are recognized risk factors for brain toxicity. From the review of published data, Lawrence et al. [63] could conclude that the incidence and severity of brain side effects is dose and volume dependent. Brain necrosis incidence of 5% occurs for BED_(*α*/*β*=  3)_ = 120 Gy, if standard fractionation regimes are employed. Experiences with single fraction radiosurgery clearly demonstrate a correlation between target size and side effects. The study of Debus et al. [64] about 367 patients receiving photon/proton radiation therapy for skull base chordoma and chondrosarcoma found a 10-year brainstem toxicity-free survival of 88%; risk factors at multivariate analysis were diabetes, number of surgical procedures, and volume of brainstem receiving 60 Gy (RBE) (*P* < 0.001). Schlampp et al. [65] observed 10 temporal lobe reactions at MRI out of 59 patients treated with carbon ions for chordoma and chondrosarcoma of the skull base. Most important predictors of toxicity were age and maximum dose applied to at least 1 cm^3^ of the temporal lobe. Carbon ions were found by Miyawaki et al. [49] to be significantly more frequently associated than protons with brain toxicity. According to the author, a reason could be found in the different fractionation schedules employed: single-fraction carbon ion dose of 3.6 Gy (RBE) would be more toxic than a proton one of 2.5 Gy (RBE). Some studies in the literature support this hypothesis as, for example, the report of Lee et al. [66] about 1008 patients treated for nasopharyngeal carcinoma: significantly higher incidence of temporal lobe necrosis was associated with single fraction size of 4.2 Gy. Lawrence et al. [63] in their review could not make any definitive conclusion about incidence and severity of toxicity when fractionated radiation therapy with single doses >2.5 Gy is used. Also in this case, the effect of the higher RBE of carbon ion cannot be ruled out definitely. 

## 5. Conclusions

Particle radiation therapy is a promising tool for the treatment of paranasal and nasal cavity malignancies. Considering that it is in its early phases of development, it has shown to be feasible and well tolerated. Results regarding local control are encouraging and late toxicity is acceptable. Further research is needed to establish its exact role and its best combination with surgery and chemotherapy.

## Figures and Tables

**Table 1:** Patient, tumor, and treatment characteristics of reports regarding patients with sinonasal malignancies treated with protons or ions.

Reference	Years	Patients, Gender	Median age in years (range)	Pathology	Stage	Surgery	Chemotherapy	Radiation therapy	F/U months (range)
Castro et al., 1994 [40]	1977–1992	22 M/F na	na	na	All with skull base invasion	na	na	Full Particle: 75% Mixed Ph/Pr: 25%	51 (4–191)^1^

Tokuuye et al., 2004 [42]	1983–2000	11 6 M/5 F	67 (40–78)	SCC: 5 MM: 2 Others: 4	IV: 4 II: 1 III: 1 Rec/na: 6	3 nos	3 nos	Full Hadron: 4/11 Mixed Ph/Pr: 7/11	23 (6–58)

Mizoe et al., 2004 [31]	1994–1997	10 M/F na	na	na	All locally advanced	All bx	na	Full Particle	90 (77–108)^1^

Resto et al., 2008 [43]	1991–2002	102 53 M/49 F	50 (15–82)	SCC: 33 CNE: 30 ACC: 20 Others: 19	All locally advanced	CR: 20 PR: 50 Bx: 32	30: Ind. EPD/CDDP 4: Other	Mixed Ph/Pr: 100%	43.2 (1.32–156.6)

Zenda et al., 2011 [44]	1999–2006	39 22 M/17 F	57 (22–84)	SCC: 11 ONB: 9 MM: 6 Others: 13	All T4N0M0	All Bx	Ind.: 10 Con.: 1	Full Particle	45.4 (1.3–90.9)

Malyapa et al., 2010 [39]	2007–2009	38 M/F na	na	na	All with skull base extension	R0: 18 R+: 17 Bx: 3	na	Full Particle	13.1 (1–29)

^1^Data refers to a wider group of head and neck patients included in the same study. SCC: squamous cell carcinoma, MM: malignant melanoma, ACC: adenoid cystic carcinoma, CNE: carcinoma with neuroendocrine features, ONB: olfactory neuroblastoma, na: not available, Rec: recurrence, nos: not otherwise specified, Ph: photon, Pr: particles, CR: complete resection, PR: partial resection, Bx: biopsy, Ind.: induction, EPD: etoposide, CDDP: cisplatin, Con.: concurrent, R0: negative resection margins, R+: positive resection margin, F/U: followup.

**Table 2:** Radiation therapy details of reports regarding patients with sinonasal malignancies treated with protons or ions.

Reference	Particle	Particle median total dose in Gy (RBE) (range)	Particle median dose per fraction in Gy (RBE) (range)	Particle fractions a week	RBE	Photon median total dose in Gy (range)	Photon median dose per fraction in Gy (range)	Median total dose in Gy (RBE) (range)
Castro et al., 1994 [40]	Helium ions	65 (60–80)	2.0 (na)	4	1.3 (1.6 CNS)	na	na	65 (60–80)

Tokuuye et al., 2004 [42]	Protons	42 (16–81)	2.5 (1.6–6.0)	na	1.0	40 (22–75)	1.8 (1.7–2)	72 (42–98)

Mizoe et al., 2004 [31]	Carbon ions	Study A^1^: 59.4 (48.6–70.2) Study B^1^: 57.6 (52.8–64.0)	Study A: 3.3 (2.7–3.9) Study B: 3.6 (3.3–4.0)	Study A: 3 Study B: 4	3.0	—	—	Study A: 59.4 (48.6–70.2) Study B: 57.6 (52.8–64.0)

Resto et al., 2008 [43]	Protons	Median proton %: 57.1 (22.9–84.8)		na	na	BID: 82 (80%)		71.6 (55.4–79.4)

Zenda et al., 2011 [44]	Protons	65 (60–70)	2.5 (2–4)	na	1.1	—	—	65 (60–70)

Malyapa et al., 2010 [39]	Protons	R0: 68.4–69.6 R+, Bx: 74.4	1.2 BID	na	na	—	—	R0: 68.4–69.6 R+, Bx: 74.4

^1^Study A: phase 1/2 dose escalation study with 10% dose increment, in 18 fractions for 6 weeks. Study B: phase 1/2 dose escalation study with 10% dose increment in 16 fractions in 4 weeks. Data from both studies refers to a wider group of head and neck patients.

RBE: relative biologic effectiveness, CNS: central nervous system, BID: bis in die, R0: negative resection margins, R+: positive resection margin, Bx: biopsy, na: not available.

**Table 3:** Results for reports regarding patients with sinonasal malignancies treated with protons or ions.

Reference	Median total dose Gy (RBE) (range)	Local control	Regional control	Distant metastasis free survival	Overall survival	Toxicity	Median time to toxicity (months)
Castro et al., 1994 [40]	65 (60–80)	5-years KM: 60% (All definitive RT)	na	na	5-years KM: 38%	na	na

Tokuuye et al., 2004 [42]	72 (42–98)	9/11^1^ (All definitive RT)	na	na	5/11^1^	Osteoradionecrosis: 1	12

Mizoe et al., 2004 [31]	Study A: 59.4 (48.6–70.2) Study B: 57.6 (52.8–64.0)	5-years KM: 49% (All definitive RT)	na	na	na	Study A^2^, Acute G3: Skin: 1 Mucosal: 0 Study B^2^, Acute G3: Skin: 6 Mucosal: 1 No late G3	na

Resto et al., 2008 [43]	71.6 (55.4–79.4)	5-years KM CR: 95% PR: 82% Bx: 87% (*P* = 0.32)	5-years KM CR: 87% PR: 88% Bx: 90% (*P* = 0.68)	5-years KM CR: 95% PR: 69% Bx: 52% (*P* = 0.03)	5-years KM CR: 90% PR: 53% Bx: 49% (*P* = 0.02)	na	na

Zenda et al., 2011 [44]	65 (60–70)	1-year KM: 77% (All definitive RT)	34/39^1^	30/39^1^	5-years KM: 55%	No severe acute Late G3^3^: Cataract: 1 Visual decrease: 1 CN VI palsy: 1 Bone necrosis: 1 Late G5: CSF leakage: 1	35.1 (range: 4.1–61.2)

Malyapa et al., 2010 [39]	R0: 68.4–69.6 R+, Bx: 74.4	36/38^4^	—	34/38^4^	—	Mild retinopathy: 1	na

na: not available, CR: complete resection, PR: partial resection, Bx: biopsy,

^1^Number of patients, ^2^Data refers to a wider group of head and neck patients, acute toxicity scored according to RTOG, late according to RTOG/EORTC, ^3^CTCAE v.3.0, ^4^Number of patients; results according to type of treatment, that is, definitive or postoperative RT not available.

**Table 4:** Treatment details of studies focusing on toxicity.

Reference	Patients	Treatment history	RBE	^1^Radiotherapy details	Median F/U in months (range)	Toxicity evaluated	Diagnostic criteria
Jensen et al., 2011 [45]	Full C: 4 Mix. C/Ph: 25	RT: 17 Relapse: 9 Re-RT: 2	na	Full C, F/SF: 15–20/3 Mix, TD/F/SF/FW: C: 24/8/3/5 P: 50/25/2/5	5.1 (2.4–10.8)	Any Acute	Physical examination

Demizu et al., 2009 [46]	C: 10 P: 42	Surgery/CMT: 4/0 Surgery/CMT: 4/12	C: 2–3.7 P: 1.1	Carbon, TD/F/SF/FW: 57.6/16/3.6/5 Proton, TD/F/SF/FW: 65/26/2.5/5	^2^C: 28 ^2^P: 25	Radiation-induced optic neuropathy	MRI CFF

Hasegawa et al., 2006 [47]	C: 14	na	na	TD: 56 F: 16–18 SF: 3.0–4.0 Weeks: 4–6	na	Radiation-induced optic neuropathy	MRI VEP

Weber et al., 2006 [48]	P/Ph: 36	Biopsy: 8 GTR: 28 CMT: 14 (no conc.)	1.1	TD: 69.6 (60.8–77) (20–84.4% protons) P, SF: 1.6 (1.4–1.8) Ph, SF: 1.8 (1.6–2.0) BID fractionation	52.4 (17–122.8)	Any late visual	Neuro-ophthalmologic evaluation

Miyawaki et al., 2009 [49]	C: 5 P: 23	All had biopsy or STR	na	Carbon: 57.6/16/2.5 Proton TD/F/SS: 65/26/2.5	^2^C: 32 ^2^P: 39	Radiation-induced brain changes	MRI Physical examination

^1^Doses expressed in Gy (RBE), numbers are median values or range if separated by a tract, ^2^Data refers to a wider group of head and neck patients, F/U: followup, C: carbon ion, Mix.: mixed, RT: radiotherapy, Re-RT: re-irradiation, Ph: photons, P: protons, CMT: chemotherapy, TD: total dose, F: number of fractions, SF: single fraction dose, FW: fractions a week, CFF: critical flicker frequency, VEP: visual evoked potential, na: not available, GTR: gross total resection, STR: subtotal resection, conc.: concomitant, BID: *bis in die.*

**Table 5:** Results of studies focusing on toxicity.

Reference	Toxicity	Median time (months, range)	Grade	D_max_ to affected OARs, Gy (RBE) (median, range)	Risk factors
Jensen, et al., 2011 [45]	7/29	na	^1^G3 mucositis: 5 ^1^G3 dysphagia: 2	na	na

Demizu et al., 2009 [46]	Carbon: 1/10 Proton: 4/42^2^	C: 52 P: 34.5 (26–39)	Counting fingers or more severe	ON: 129^3^ ON: 118^3^, (61–126)	^4^UA: Age > 60 years; DM, D_max_> 110 Gy (RBE) ^4^MA: DM

Hasegawa et al., 2006 [47]	7/14^5^	24 (10–41)	Complete visual loss	ON: 57.6, (57.6–64)	^4^UA: MG, CMT, anemia, DM, TD, D_max_ to ON, D_10-50_, MA: D_20_ > 60 Gy (RBE)

Weber et al., 2006 [48]	13/36 5-years ≥G2: 20.7% ± 7.8	31.5 (6.4–91)	^6^G1: 5 G2: 6 G3: 2 (CA, NLB)	ON: 54.7, (47.8–80) OC: 52.1, (26.2–56.4)	GTV Dose OC: D_90_ and D_50 _ ON: D_10 _ Younger Age

Miyawaki et al., 2009 [49]	C: 2/5 P: 3/23	C: 27.5 (19–36) P: 31 (6–49)	^7^C: G1: 1, G3: 1 ^7^P: G1, G2, G3: 1	C, D_min_: 27.5^3^ (102.4–110.6) P, D_min_: 117.1^3^ (59.4–117.1)	^4^Carbon ions, Dose ≥ 80 Gy (RBE)^3^, Volumes > 83, 90, 100 Gy (RBE)^3^

^1^CTCAE versus 4.0, ^2^One patient experienced bilateral vision loss, ^3^Doses are expressed in biologically equivalent dose (BED) at *α*/*β* = 3, ^4^Data refer to a wider group of patients with head and neck malignancies, ^5^Two patients had bilateral visual loss, ^6^Dry-eye syndrome and epiphora were evaluated with CTCAE v.2.0, all the others with LENT/SOMA, ^7^CTCAE v. 3.0, OARs: organs at risk, C: carbon ions, P: protons, na: not applicable, ON: optic nerve, OC: optic chiasm, DM: diabetes mellitus, MG: male gender, TD: tumor dose, CMT: chemotherapy, UA: univariate analysis, MA: multivariate analysis, CA: cataract, NLB: nasolacrimal duct blockage, D_10, 20, 50, 90_: dose to the 10%–20%–50%–90% of the organ considered.

**Table 6:** Outcome results for studies employing photon radiation therapy.

Author, institution, year	Treatment, patients	Median total dose Gy (range)	LC 5-yrs KM	RC 5-yrs KM	DC 5-yrs KM	OS 5-yrs KM	Toxicity
Mendenhall et al., UF, 2009 [10]	Pre-op RT: 8 Post-op RT: 45 Def RT: 56	Pre-op RT: 55 (48.4–64.8) Post-op RT: 64.8 (55–74.4) Def RT: 70 (50–70)	Adj.: 84% Def: 43% (*P* = 0.0007)	N0: 93% N1: 88%	81%	Adj: 73% Def: 38% (*P* < 0.0001)	Late ≥ G3 Def: 17 (30%) Late ≥ G3 Pre-op: 1 (12.5%) Late ≥ G3 Post-op: 12 (26.7%)

Chen et al., UCSF, 2007 [24]	Pre-op RT: 9 Post-op RT: 82 Def RT: 36	Pre-op RT: 60 Post-op RT: 63 Def RT: 66	GTR: 65% GRD: 44% (*P* = 0.02)	^1^120/127	^1^113/127	52%	Late ≥ G3: (*P* < 0.01) 53% (1960s), 45% (1970s), 39% (1980s), 28% (1990s), 16% (2000s)

Daly et al., UCSF, 2007 [50]	Post-op IMRT: 32 Def IMRT: 4	Post-op IMRT: 58 (51–60) Def IMRT: 70 (63–72)	58%	^1^35/36	^1^31/36	45%	Acute ≥ G3: 7 (19.4%) Any Late: 12 (40%)

Hoppe et al., MSKCC, 2008 [21]	Def RT: 39	70 (48–72) (BED)	21%	61%	51%	15%	Acute ≥ G3: 20 (51.3%) Late ≥ G3: 7 (18%)

Hoppe et al., MSKCC, 2007 [20]	Post-op RT: 85	63 (50–70)	62%	87%	82%	67%	Acute ≥ G3: 18 (21%) Late ≥ G3: 1 (2.5%)

Madani et al., Ghent University Hospital, 2009 [51]	Post-op IMRT: 75 Def IMRT: 9	Post-op/Def: 70 (48–72)	70.7%	—	82.2%	58.5%	Acute G3: 6 (7.1%) Late ≥ G3: 8 (9.5%)

Dirix et al., Leuven University Hospital, 2010 [52]	Post-op IMRT: 40	60 (60–66)	76% (2-yrs)	100% (2-yrs)	89% (2-yrs)	89% (2-yrs)	No Acute ≥ G3 No Late ≥ G3

^1^Number of patients, LC: local control, RC: regional control, DC: distant control, OS: overall survival, KM: Kaplan-Meier, Pre-op RT: preoperative radiotherapy, Post-op RT: postoperative radiotherapy, Def RT: definitive radiotherapy, Adj: pre-op and post-op radiotherapy, GTR: gross tumor resection, GRD: gross residual disease, IMRT: Intensity Modulated Radiation Therapy, BED: Biological Effective Dose, UF: University of Florida, UCSF: University of California San Francisco, MSKCC: Memorial Sloan-Kettering Cancer Center.
